# Large Language Models Using Clinical Text in Pediatrics: A Scoping Review

**DOI:** 10.1001/jamanetworkopen.2026.2443

**Published:** 2026-03-25

**Authors:** Tracy Huang, Gabriel Tse, Natalie M. Pageler, Yair Bannett

**Affiliations:** 1 Division of Developmental-Behavioral Pediatrics, Stanford University School of Medicine, Stanford, California; 2 Department of Pediatrics, Stanford University School of Medicine, Stanford, California; 3 Division of Clinical Informatics, Department of Pediatrics, Stanford University School of Medicine, Stanford, California

## Abstract

**Importance:**

Large language models (LLMs) are increasingly being applied to analyze clinical data, primarily clinical text, with an increasing emphasis on integration in health care. However, the use of LLMs in pediatric care remains underexplored.

**Objective:**

To map the emerging literature on LLM use in pediatrics involving clinical text and identify evidence gaps and future directions for implementation and evaluation.

**Evidence Review:**

PubMed/MEDLINE, Embase, Web of Science, Scopus, and preprint servers were searched for English-language original research published from January 1, 2020, to July 1, 2025. Included studies used modern transformer-based LLMs with pediatric clinical text as input. Two reviewers independently screened studies using predefined criteria. Data were extracted by one reviewer and verified by another. Findings were descriptively synthesized, and adherence to the Minimum Information for Medical AI Reporting (MINIMAR) standards was assessed.

**Findings:**

The review included 40 studies published between 2023 and 2025. Twenty-three studies were conducted in the US, and all were retrospective observational studies using clinical data from sources such as electronic health records. Participant sample sizes ranged from 10 to 172 683. Although all pediatric age subgroups were represented, early childhood populations (aged 0-5 years) were underrepresented. The most common LLM clinical applications were diagnostic decision support in 24 studies (60.0%) and treatment planning in 7 studies (17.5%). Although all 40 studies conducted clinical evaluation of LLMs and 30 included discussions of ethics or data privacy, 39 studies (97.5%) did not meet full MINIMAR standards, 34 (85.0%) did not report use of Health Insurance Portability and Accountability Act–compliant models, and 30 (75.0%) lacked fine-tuning for pediatric-specific data. Among 33 studies assessing model performance against human annotations, 10 (30.3%) did not include clinicians as annotators; among 26 studies with multiple annotators, only 9 (34.6%) reported interannotator agreement statistics.

**Conclusions and Relevance:**

This scoping review found that diagnostic decision support and treatment planning were commonly proposed applications of LLMs in pediatrics. However, gaps in scientific rigor and limited use of pediatric-specific data may hinder their safe and effective implementation in pediatrics. Future studies should use standardized evaluation and reporting methods, increase clinician involvement, and expand research to underrepresented ages and clinical applications.

## Key Points

**Question:**

How are large language models (LLMs) being used to analyze clinical text in pediatrics?

**Findings:**

This scoping review of 40 studies, all published within the past 2 years, found diverse clinical applications of LLMs, most commonly for clinical decision support, across multiple pediatric subspecialties. However, there was limited use of transparent reporting, standardized evaluation methods, and ethical or data privacy safeguards.

**Meaning:**

This study’s results suggest that it is imperative to prioritize pediatric-specific data and adherence to rigorous reporting and evaluation standards to ensure safe and effective implementation of LLMs for analyzing clinical text in pediatrics.

## Teaser

This scoping review examines research on large language model use in pediatric clinical text analysis.

## Introduction

Large language models (LLMs) are a new class of artificial intelligence (AI) tools enabled by advances in computing power, large-scale training datasets, and novel model architectures.^1^ Unlike earlier pretrained language models that required task-specific fine-tuning, modern LLMs are general-purpose foundation models capable of performing diverse tasks across multiple domains, often without additional training.^1^ They demonstrate humanlike abilities in understanding, reasoning, and generating text. The Google T5 (Text-to-Text Transfer Transformer) introduced a unified text-to-text framework for natural language processing tasks, inspiring subsequent models with greater scale, broader data, and stronger generalization.^2^ This technology has prompted a rapid increase in studies exploring LLM applications in health care, with an increasing focus on LLM analysis of clinical text, a domain with high potential for direct impact on clinical care.^3^

Several reviews have examined LLM use in health care,^4,5^ particularly with clinical text, such as electronic health records (EHRs).^6^ However, they often do not distinguish adult from pediatric care nor focus on pediatric applications. Existing pediatric LLM reviews are limited to specific tools (eg, ChatGPT [OpenAI])^7^ or narrow topics, such as patient education.^8^ To our knowledge, no comprehensive review has evaluated modern LLMs using clinical data in pediatrics. Such a review is particularly important because pediatric-focused applications present unique challenges, including distinct clinical presentations, developmental variability, pediatric-specific subspecialties, and broader stakeholder involvement (eg, parents and caregivers).^9^ Ethical, privacy, and data-sharing concerns further complicate pediatric LLM use because children’s health data are subject to strict consent and protection requirements. Additionally, general-domain LLMs trained on adult or nonclinical data may underperform or introduce bias in pediatric contexts.^9^

This scoping review addresses a critical gap by mapping research on LLM use in pediatric clinical text analysis and identifying evidence gaps to guide future research, implementation, and evaluation of pediatric LLM applications. We focused on LLMs developed after T5 to capture technologies relevant to current clinical practice^2^ and on studies using clinical documentation rather than synthetic or fictionalized inputs to emphasize translational relevance of LLMs in clinical settings.^4^

## Methods

This scoping review followed the Arksey and O’Malley^10^ framework, as revised by Colquhoun et al^11^ and adhered to Preferred Reporting Items for Systematic Reviews and Meta-Analyses Extension for Scoping Reviews (PRISMA-ScR) reporting guidelines. The protocol was registered on Open Science Framework before study selection. This review was exempt from institutional review board approval because it used only publicly available data.

### Literature Search Strategy

We searched PubMed/MEDLINE, Embase, Web of Science, Scopus, and preprint servers (arXiv, medRxiv, and Research Square) using LLM- and pediatric-related terms (eg, *large language model*,* generative artificial*,* pediatrics*). The search covered publications from January 1, 2020, to July 1, 2025, with publication type filters applied a priori to limit retrieval to empirical studies. Articles published online within the search period were included, regardless of formal publication date. One additional eligible article identified by manual search on July 7, 2025, was included because it met predefined criteria before screening. The full search strategy is provided in eTable 1 in Supplement 1.

### Study Selection and Eligibility Criteria

After deduplication, 2 reviewers (T.H. and G.T.) independently screened titles and abstracts as well as full texts using standardized forms in Covidence (Veritas Health Innovation), with disagreements resolved by consensus or third-reviewer adjudication (Y.B.). We applied the following inclusion criteria: (1) LLM type: use of at least 1 post-T5 generative, transformer-based LLM trained on large-scale corpora with capability for multitask generalization; (2) data source: clinical text data routinely generated during clinical care (eg, EHR data and clinical notes) as LLM input; (3) population: pediatric patients (aged 0-18; studies including pediatric subgroups or populations extending to young adults [aged ≤24 years] were included if pediatric patients were represented); and (4) use case: application of the LLM to a clinical or research task rather than a purely methodological evaluation. We included English-language original research articles, research letters, preprints, and full-text conference proceedings. Letters to the editor were excluded unless they reported original data. No restrictions were placed on study design, geographic location, or LLM application.

### Data Extraction and Synthesis

Data were extracted using a piloted custom form by a first reviewer (T.H.) and verified by a second reviewer (G.T.), with discrepancies resolved by consensus or third-reviewer adjudication (Y.B.). Data were synthesized using descriptive statistics and narrative summaries in Excel, version 16.105 (Microsoft Corp) (eTable 2 in Supplement 1). Reported benefits and limitations were extracted verbatim from author-reported Results and Discussion sections. LLM use cases were categorized using the validated clinical task taxonomy by Bedi et al.^12^ To assess adherence to AI reporting standards in pediatric LLM studies, we used the Minimum Information for Medical AI Reporting (MINIMAR) framework because it provides general principles for describing population, data sources, model development, and evaluation across a broad range of medical AI tasks.^13^ MINIMAR adherence was assessed to describe reporting completeness rather than methodological quality. For items related to optimization and internal model validation, adherence was evaluated only in studies that trained or fine-tuned an LLM.

## Results

### Study Characteristics Overview

**Figure 1.**
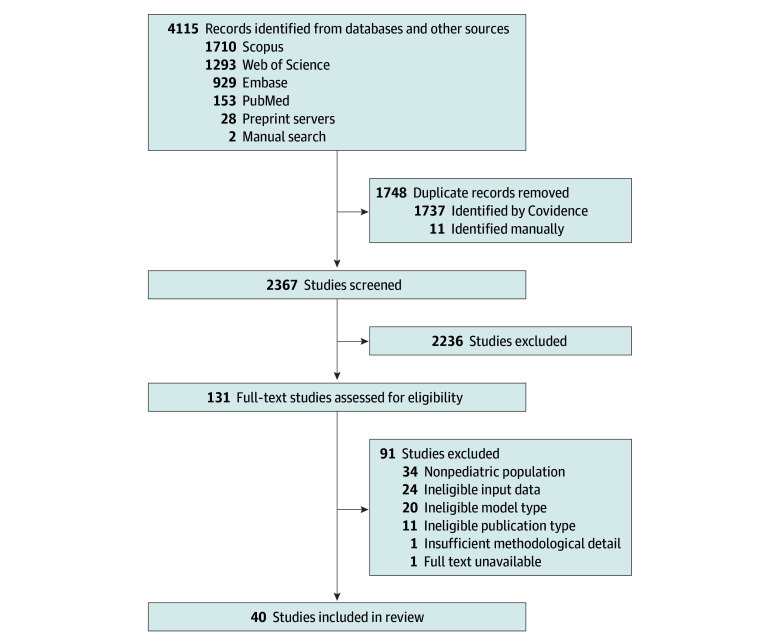
Flowchart of Included Studies

Forty studies^14,15,16,17,18,19,20,21,22,23,24,25,26,27,28,29,30,31,32,33,34,35,36,37,38,39,40,41,42,43,44,45,46,47,48,49,50,51,52,53^ met the inclusion criteria (Figure 1). All were published between 2023 and 2025, with 21 studies^14,16,17,18,22,23,24,25,26,29,30,33,34,35,36,37,39,41,43,47,49^ (52.5%) appearing in 2024 and 16 studies^15,19,20,27,28,31,32,38,40,42,44,45,48,51,52,53^ (40.0%) in 2025. Key characteristics of all included studies were reported, grouped by pediatric subspecialty (eTable 3 in Supplement 1). Twenty-four studies^14,15,16,19,20,21,23,25,28,30,31,32,33,35,38,39,40,41,42,44,45,46,48,53^ (60.0%) were original research articles, 5 studies^36,37,43,49,50^ (12.5%) were brief reports or research letters, 7 articles^17,22,26,27,29,47,52^ (17.5%) were preprints, and 4 articles^18,24,34,51^ (10.0%) were conference proceedings. All studies were retrospective observational analyses of existing clinical records, with data collection ranging from 2002 to 2024 (mean [SD], 5.3 [5.2] years). Geographically, 23 studies^16,19,20,22,23,24,25,26,27,28,32,34,36,40,41,42,43,44,45,49,51,52,53^ (57.5%) were conducted in the US and 4 studies^29,31,47,50^ (10.0%) in China. The remaining 13 studies^14,15,17,18,21,30,33,35,37,38,39,46,48^ (32.5%) were conducted across 11 countries spanning Europe, the Middle East, North Africa, and Asia.

### Pediatric Population Characteristics and Sample Size

Nearly all studies focused on pediatric populations, but 2 studies^25,52^ (5.0%) included pediatric data alongside adult datasets. Nineteen studies^14,19,20,21,22,23,26,27,31,32,34,35,38,39,40,41,48,49,51^ (47.5%) reported any age-related statistic (eg, mean, median, or range), but 24 studies^15,16,17,18,23,24,25,26,28,29,30,32,33,36,37,42,43,44,45,46,47,50,52,53^ (60.0%) did not specify pediatric subgroup (eg, adolescents) ages. Among studies that reported specified age subgroups, only 9 studies^20,21,26,30,31,33,34,48,53^ (22.5%) included young children (birth to 5 years). Pediatric sample sizes ranged from 10 to 172 683, with ages spanning birth to 18 years. However, 7 studies^27,28,29,35,39,42,44^ (17.5%) used small samples (<50 patients).

### Study Settings and Data Sources

Clinical settings included 15 academic freestanding children’s hospitals (37.5%), 13 pediatric units within a general or academic adult hospital (32.5%), 9 community-based clinics or health care networks (22.5%), and 3 research repositories or data collaboratives (7.5%). Twenty-five studies^15,18,19,20,22,23,27,29,31,32,33,34,35,36,37,40,41,43,45,46,48,49,50,51,52^ (62.5%) were conducted in outpatient settings, 11^14,16,21,25,26,28,30,38,39,42,53^ (27.5%) in inpatient settings, 2^17,47^ (5.0%) in combined inpatient and outpatient settings, and 2^24,44^ (5.0%) in unspecified settings. No studies assessed LLMs that were integrated into pediatric clinical workflows.

Nearly all studies used exclusively observational pediatric patient data, with 2 studies^34,36^ (5.0%) also incorporating nonclinical data from parents or caregivers. Clinical text data sources for LLM analysis included clinical notes in 13 studies^16,18,19,20,23,27,30,37,43,46,48,51,52^ (32.5%), diagnostic or imaging reports in 7 studies^14,21,25,26,28,33,53^ (17.5%), contextual patient data in 5 studies^24,36,41,44,45^ (12.5%), structured or semistructured EHR data in 3 studies^31,38,49^ (7.5%), audio-transcribed interactions in 2 studies^22,29^ (5.0%), behavioral or developmental assessments in 1 study^34^ (2.5%), and composite EHR datasets that combined 2 or more of the above data categories in 9 studies^15,17,32,35,39,40,42,47,50^ (22.5%). Data sources were in English in 27 studies^14,16,19,20,22,23,24,25,26,27,28,31,32,34,35,36,38,40,41,42,43,44,45,49,51,52,53^ (67.5%). Other languages included Chinese, Turkish, French, Thai, Hebrew, Spanish, and Italian.

### LLM Characteristics and Fine-Tuning

The most studied LLM was GPT (OpenAI) in 29 studies^14,15,16,17,20,21,23,24,25,28,29,30,31,32,33,35,36,37,38,39,40,41,42,43,44,46,48,49,50^ (72.5%) followed by LLaMA (Meta) in 9 studies^16,19,22,27,29,45,47,51,52^ (22.5%). Twenty-three studies^14,15,17,18,19,20,23,25,26,33,35,36,37,39,40,41,42,43,44,46,48,49,50^ (57.5%) evaluated a single LLM, whereas the remaining 17 studies^16,21,22,24,27,28,29,30,31,32,34,38,45,47,51,52,53^ (42.5%) compared multiple models. Thirty studies^14,17,18,20,21,22,23,25,27,28,29,30,31,32,33,34,35,36,37,38,39,41,42,43,44,46,48,49,50,52^ (75.0%) relied solely on pretrained models, 6 studies^19,26,40,45,51,53^ (15.0%) fine-tuned the LLMs on pediatric data, and 4 studies^15,16,24,47^ (10.0%) used both fine-tuned and pretrained models.

### Clinical Applications and Pediatric Subspecialties

**Figure 2.**
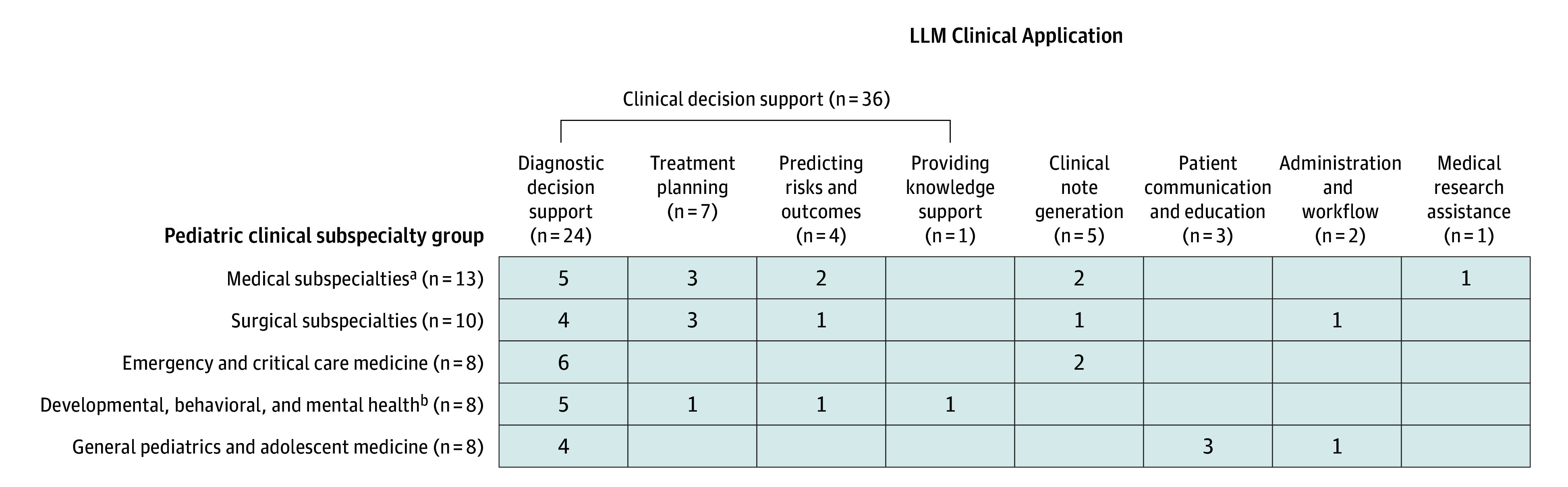
Matrix of Large Language Model (LLM) Clinical Applications by Pediatric Clinical Subspecialties This figure maps LLM use case categories and subcategories, based on validated taxonomy,^12^ across pediatric subspecialty groups. Studies addressing multiple use cases were counted in each relevant category. ^a^Pediatric medical subspecialties include fetal cardiology, pediatric clinical genetics, infectious diseases, nephrology, neurology, and oncology. ^b^Developmental, behavioral, and mental health includes developmental-behavioral pediatrics and child and adolescent psychiatry.

Figure 2 summarizes LLM clinical applications across pediatric subspecialties. Clinical decision support was the most studied category, followed by clinical note generation, patient communication and education, administration and workflow, and medical research assistance. Two studies^42,47^ (5.0%) included multiple applications. Twelve distinct subcategories of LLM applications were identified, with diagnostic decision support in 24 studies^14,15,16,17,18,20,21,22,24,27,29,32,33,35,37,39,40,41,42,46,47,50,52,53^ (60.0%) and treatment planning in 7 studies^31,34,35,39,42,47,51^ (17.5%) being the most common. Examples of diagnostic decision support included generating differential diagnoses for pediatric intensive care unit patients^16^ and recommending cancer predisposition genes for pediatric patients with cancer.^46^ Examples of treatment planning included optimizing surgical procedure timing for pediatric cardiovascular patients^42^ and determining early intervention for childhood refractive error.^31^

The 18 represented pediatric subspecialties were grouped into pediatric medical specialties; emergency medicine and critical care; developmental, behavioral, and mental health; general pediatrics and adolescent medicine; and surgical specialties. The most common application-subspecialty intersection was diagnostic decision support in emergency and critical care medicine.^15,16,20,37,41,53^

### LLM Evaluation

All studies evaluated LLM performance. Fifteen studies^15,18,20,21,22,27,28,30,31,34,36,45,51,52,53^ (37.5%) compared evaluation metrics across different LLMs or between LLMs and other machine learning or AI models, and 9 studies^19,25,26,32,37,38,41,46,47^ (22.5%) evaluated a single LLM. Common evaluation metrics included accuracy in 20 studies^14,15,17,21,23,25,26,31,33,37,39,40,42,46,47,48,49,50,51,52^ (50.0%), F1 score in 12 studies^14,15,18,22,24,27,30,32,34,40,45,53^ (30.0%), sensitivity in 9 studies^19,20,21,26,36,37,40,43,48^ (22.5%), and specificity in 8 studies^19,20,21,26,37,40,43,48^ (20.0%). Thirty-three studies^14,15,16,17,18,19,20,21,22,23,24,25,26,27,29,30,31,33,34,35,36,37,39,40,42,43,44,47,48,49,50,51,53^ (82.5%) assessed model performance against human-annotated ground truth labels. Among those, 22 studies^14,15,16,17,19,20,21,23,26,29,35,36,37,39,40,42,43,47,49,50,51,53^ (66.7%) used clinician annotators, 10 studies^18,22,24,25,30,31,33,34,44,48^ (30.3%) used trained nonexperts (eg, medical students and researchers), and 1 study^27^ (3.0%) did not provide annotator details. Multiple annotators were used in 26 studies^15,16,17,18,19,20,21,22,24,25,26,27,30,31,33,34,35,36,40,43,44,48,49,50,51,53^ (65.0%), but interannotator agreement was reported in only 9 such studies^16,19,20,21,24,26,44,48,49^ (34.6%).

### Benefits and Limitations Reported

Studies reported measurable benefits, including improved accuracy, time efficiency, and cost-effectiveness, along with limitations, including hallucinations and inconsistent performance; these author-reported benefits and limitations are summarized in Table 1. A commonly cited benefit of LLMs was analyzing unstructured clinical text at scale. Common study design limitations included limited generalizability, retrospective designs, small sample sizes, data quality and missingness, suboptimal interrater agreement, and lack of task-specific fine-tuning.

**Table 1.** Pediatric-Specific LLM Benefits and Limitations

Benefit or limitation	Description	Relevance to pediatric settings	No. of studies that mentioned benefits	Affected use case categories and subcategories
**Pediatric-relevant benefits**				
Strong model performance	LLMs demonstrate strong performance and/or are comparable to or exceeding that of baselines or human experts in clinical metrics, including accuracy, sensitivity, specificity, positive predictive value, negative predictive value, and F1 score	Improved performance in pediatric tasks helps clinicians identify rare diseases, assess developmental milestones more precisely, and recommend safer treatments, all areas where both false-positive and false-negative findings can have lasting clinical consequences	27^14,15,17-22,24-28,30-32,34,36,37,40,45-48,51-53^	Administration and workflow (overseeing financial activities) Clinical decision support (diagnostic decision support, predicting risks and outcomes, providing clinical knowledge support, treatment planning) Clinical note generation (documenting care plans, documenting diagnostic reports, documenting patient visits) Patient communication and education (patient-practitioner messaging)
Time efficiency	LLMs reduce time spent on clinical, research, or documentation tasks	Time efficiency allows clinicians to spend more time on other tasks, such as patient interaction, better caregiver communication, and faster documentation in critical settings, such as NICUs and emergency departments	3^14,15,25^	Clinical decision support (diagnostic decision support, predicting risks and outcomes) Clinical note generation (documenting diagnostic reports)
Cost-saving measures	LLMs lower cost of labor, infrastructure, or manual processes	Reducing costs in pediatric care expands access in underresourced areas and frees funds for specialists, therapies, and equipment	3^15,25,32^	Clinical decision support (diagnostic decision support) Clinical note generation (documenting diagnostic reports)
Consistent performance	LLMs have stable performance metrics across evaluations with low variability	Consistent performance metrics across evaluations ensure that LLM outputs are stable and reproducible, supporting reliable clinical assessments, consistent decision-making, and equitable care across patients, practitioners, and clinical scenarios	4^17,32,40,53^	Clinical decision support (diagnostic decision support)
Domain-specific fine-tuning improves performance	Fine-tuned LLMs outperform general-purpose LLMs in medical use or have strong performance	Fine-tuning on pediatric data improves model accuracy for areas such as age-specific diagnoses, development assessments, and medication dosing, especially where general models often fall short	9^15,16,19,24,40,45,47,51,53^	Clinical decision support (diagnostic decision support, predicting risks and outcome, providing clinical knowledge support, treatment planning) Clinical note generation (documenting care plans)
Scalable automation	LLMs enable processing of large-scale, unstructured data with minimal human input; scalable automation is defined as LLM use on unstructured pediatric datasets of approximately ≥500 records or notes in the evaluation set (not training or validation), with minimal manual processing	Scalable automation supports efficient analysis of extensive pediatric clinical records, enabling large-scale research, surveillance, and timely identification of health trends with reduced manual effort	8^15,20,26,32,36-38,41^	Clinical decision support (diagnostic decision support, predicting risks and outcomes, clinical note generation, documenting diagnostic reports) Patient communication and education (patient-practitioner messaging)
Adaptability to other languages	LLMs perform well on non-English or underrepresented linguistic data	Language adaptability is crucial in pediatrics, where effective communication with children and families from diverse linguistic backgrounds improves equity, reduces miscommunication, and enhances care quality across multicultural populations	9^15,17,18,29,30,37,44,47,48^	Clinical decision support (diagnostic decision support, predicting risks and outcomes, treatment planning) Clinical note generation (documenting care plans, documenting patient visits) Patient communication and education (enhancing accessibility)
**Pediatric-relevant limitations**				
Hallucinations	LLM fabricates facts or clinical content	Hallucinated content poses serious risks in pediatrics, where inaccurate diagnoses, fabricated dosing information, or false reassurance can directly impact vulnerable patients’ safety and long-term development	7^22-25,43,51,53^	Administration and workflow (organizing workflow processes) Clinical decision support (diagnostic decision support, treatment planning) Clinical note generation (documenting diagnostic reports) Medical research assistance (analyzing research data)
Inconsistent performance	LLM produces variable results across similar prompts or tasks	Inconsistent outputs can undermine trust and reliability in high-stakes pediatric care, leading to variability in clinical decisions, parental guidance, or developmental assessments across similar cases	3^29,39,42^	Clinical decision support (diagnostic decision support, treatment planning) Clinical note generation (documenting patient visits)
Lack of transparency or verifiable sources	LLM fails to cite sources or cites incorrect or fabricated ones	Lack of reliable references limits clinicians’ ability to verify LLM-generated content, especially critical in pediatrics, where evidence-based care must be tailored to age, weight, and developmental stage	2^21,50^	Clinical decision support (diagnostic decision support)
Difficulty with processing complex data	LLM struggles with interpreting multistep, nuanced, or high-volume clinical data	Pediatric cases often involve layered data, such as developmental history, growth metrics, family context, and age-specific norms, making it essential for LLMs to accurately process complex, nuanced information to avoid misinterpretation or oversight	10^23,29-31,40,42,46,50-52^	Clinical decision support (diagnostic decision support, treatment planning) Clinical note generation (documenting patient visits) Medical research assistance (analyzing research data)
Prompt sensitivity	LLM’s output quality depends heavily on how the prompt is phrased or structured	High prompt sensitivity limits reliability in busy pediatric environments, where clinicians and caregivers may use varied, informal, or incomplete language	7^14,18,24,42,48,51,52^	Clinical decision support (diagnostic decision support, predicting risks and outcomes, treatment planning) Clinical note generation, documenting patient visits)
Insufficient pediatric or domain-specific understanding	LLM lacks sufficient training in pediatric or other specialty domains and misreads or confuses medical abbreviations, jargon, or terminology	Pediatric care requires age-specific medical knowledge, including growth patterns, developmental milestones, and pediatric pharmacology, as well as unique terminology and abbreviations different from adult care; without this expertise, LLMs may provide unsafe or irrelevant recommendations	11^20,21,29,31,33,35,39,40,46,50,52^	Clinical decision support (diagnostic decision support, treatment planning)

Abbreviation: LLM, large language model; NICU, neonatal intensive care unit.

### Privacy and Ethical Considerations

Thirty studies^14,15,16,17,19,20,21,22,23,25,27,31,32,33,35,36,37,39,40,41,42,43,44,45,46,49,50,51,52,53^ (75.0%) discussed LLM-related data privacy or ethical issues, but 34 studies^14,15,16,17,18,19,20,21,22,23,26,27,28,29,30,31,33,34,35,37,38,39,40,42,43,45,46,47,48,49,50,51,52,53^ (85.0%) did not indicate whether Health Insurance Portability and Accountability Act (HIPAA)–compliant models were used. Six studies^14,31,32,37,38,39^ (15.0%) specifically reported using HIPAA-compliant proprietary models, and 2 studies^40,41^ (5.0%) reported local or private model deployment.

### Error and Bias Assessment

Twelve studies^19,20,21,22,23,24,25,29,30,33,44,51^ (30.0%) included error analyses, and 2 studies^40,41^ (5.0%) included subgroup analyses. One study^19^ (2.5%) assessed model bias through fairness analysis across patient subgroups, and 2 studies^41,45^ (5.0%) reported mitigation strategies to address potential model bias. No studies reported involving external stakeholders in study design, LLM development, or evaluation.

### Data Reporting Standards

Adherence to MINIMAR reporting guidelines^13^ varied across studies (Table 2). All studies reported the population, cohort selection details, and data source, and 36 (90.0%) described the study setting. However, demographic reporting was inconsistent. Model architecture details were usually described, whereas data missingness and data availability statements were less commonly reported. External validation reporting was common, and of 10 studies using fine-tuned models,^15,16,19,24,26,40,45,47,51,53^ 9 studies^15,16,19,24,40,45,47,51,53^ (90.0%) reported on optimization and 7 studies^16,19,24,40,47,51,53^ (70.0%) reported on internal validation.

**Table 2.** Studies Meeting MINIMAR Reporting Guidelines

MINIMAR reporting guideline	Guideline description	No. (%) of studies that met reporting guideline (N = 40)
**Study population and setting guidelines**		
Population	Population from which study sample was drawn	40 (100)
Study setting	The setting in which the study was conducted (eg, academic medical center, community health care system, rural health care clinic)	36 (90)
Data source	The source from which data were collected	40 (100)
Cohort selection	Exclusion and inclusion criteria	40 (100)
**Patient demographic characteristics guidelines**		
Age	Age of patients included in the study	24 (60.0)
Sex	Sex breakdown of study cohort	15 (37.5)
Race	Race characteristics of patients included in the study	6 (15.0)
Ethnicity	Ethnicity breakdown of patients included in the study	6 (15.0)
Socioeconomic status	A measure or proxy measure of the socioeconomic status of patients included in the study	3 (7.5)
**Model architecture guidelines**		
Model output	The computed result of the model	40 (100)
Target user	The intended user of the model output (eg, clinician, hospital management team, insurance company)	40 (100)
Gold standard	Labeled data used to train and test the model	40 (100)
Model task	Classification or prediction	40 (100)
Model architecture	Algorithm type (eg, machine learning, deep learning)	40 (100)
Features	List of variables used in the model and how they were used in the model in terms of categories or transformation	40 (100)
Data splitting	How data were split for training, testing, and validation	39 (97.5)
Missingness	How missingness was addressed: reported, imputed, or corrected	13 (32.5)
**Model evaluation guidelines**		
External model validation	External validation using data from another setting	39 (97.5)
Transparency	How code and data are shared with the community	21 (52.5)
Optimization	Model or parameter tuning applied	9 (90.0)^a^
Internal model validation	Study internal validation	7 (70.0)^a^

Abbreviations: MINIMAR, Minimum Information for Medical AI Reporting.

^a^ These criteria were applicable only to studies that trained or fine-tuned a large language model (n = 10). Studies evaluating fixed, pretrained large language models without investigator-led model modification (n = 30) were considered not applicable and were excluded from the denominator.

## Discussion

This scoping review mapped the literature on LLM use with pediatric clinical text and identified 40 relevant studies from 4115 records, underscoring the early stage of this research. Despite increasing interest in LLM pediatric applications, none of the included studies evaluated LLMs integrated into real-time pediatric workflows, highlighting a gap between experimental evaluation and clinical deployment. Although studies explicitly evaluating LLMs as the primary technology in pediatrics remain limited, LLM-enabled systems for clinical documentation (ie, ambient AI scribes) are increasingly used in pediatric practice.^54^ These multimodal applications represent an adjacent domain outside the scope of this review and warrant dedicated future investigation. Publication volume increased sharply from 2023 to 2025, reflecting accelerating interest in pediatric LLM applications, consistent with trends in other health care domains.^55^ All studies used retrospective, observational designs with inherent methodological limitations. Although many reported benefits, including improved accuracy, efficiency, and cost savings, common limitations, such as hallucinations, highlight the need for continued research to support safe and effective pediatric LLM implementation.

The most studied LLM clinical applications in pediatrics were clinical decision support, mainly diagnostic decision support and treatment planning, aligning with findings in a recent health care LLM review.^56^ The predominance of decision support applications observed in this review contrasts with the increasing deployment of LLM-based administrative tools (eg, clinical text documentation and summarization and drafting of patient communications) in clinical practice.^57^ Although administrative tasks represent a lower-risk and technically well-suited use case for LLMs, pediatric-focused evaluations of these tools remain limited in peer-reviewed literature, with only a few studies assessing summarization and patient communication.^28,30,36,42,43,44,49^ This finding may reflect a research emphasis on higher-impact decision support applications in which pediatric-specific clinical context is essential as well as the tendency for administrative tools to be evaluated in broader populations rather than pediatric-specific cohorts. Future studies should also evaluate low-risk administrative applications to better align research priorities with clinical adoption.

LLM clinical use cases were distributed across a range of pediatric subspecialties, including pediatric surgery, fetal cardiology, and child and adolescent psychiatry, reflecting early exploration of LLM applications in diverse clinical contexts. Despite the diversity of pediatric subspecialties represented, underrepresented pediatric subspecialties included pediatric endocrinology, gastroenterology, rheumatology, dermatology, and allergy-immunology.

Study design challenges were common. Nearly one-fifth of studies had small sample sizes, limiting statistical power and generalizability, a finding also noted in a prior systematic review of AI research in pediatric oncology.^58^ Most studies lacked disaggregated pediatric data and age-specific analyses, even when datasets could support subgroup evaluations. This finding is concerning given the wide variation in clinical presentation and health care needs across developmental stages. Future studies should leverage modern LLM capabilities to analyze large datasets with disaggregated pediatric population data, helping to overcome the longstanding scarcity of pediatric clinical trials and meta-analyses that perform age subgroup evaluations.^59^

Most studies used pretrained LLMs developed on primarily adult data, which may underperform in pediatric contexts without fine-tuning.^60^ Pediatric datasets are typically much smaller than adult datasets, raising concern that models trained predominantly on adult data may generate outputs that reflect adult rather than pediatric characteristics.^61^ This could introduce age-related bias, where LLMs deployed in pediatrics may underperform compared with adult contexts. More research is necessary to determine whether fine-tuning LLMs on pediatric data may improve LLM performance in pediatrics.

LLM evaluation methods varied widely across studies, underscoring the need for a standardized evaluation framework.^5^ Prior work shows that clinician panels can produce reliable ground-truth labels even in complex clinical scenarios.^62^ Although labeling large datasets may be impractical, incorporating clinician benchmarks on representative samples is essential for providing meaningful context and assessing applicability.^63^ Future work should prioritize using gold-standard labels, ideally annotated by expert clinicians or highly trained annotators through a standardized training process with reported interrater agreement.^64^ Comparing LLM performance to expert review can enhance clinician and patient trust and increase the likelihood of successful implementation in pediatrics.^65^

Pediatric LLM research requires explicit attention to health equity. Equity considerations are particularly important in children given their increased vulnerability, the cumulative effects of early-life disparities, and the potential for preventive intervention.^66^ AI technologies can propagate or exacerbate existing health inequities, underscoring the importance of evaluating model fairness and implementing bias mitigation strategies.^67,68^ Specifically, proprietary LLMs trained on web-scale datasets, commonly used in the included studies, have been widely reported to introduce societal biases that are difficult to mitigate.^69^ Despite these concerns, evaluation of bias in pediatric LLM studies was remarkably limited. Only 1 included study assessed model bias, and only 2 included studies described any bias mitigation approach. Subgroup analyses by age, race or ethnicity, and other patient characteristics were uncommon, limiting detection of differential performance across populations. Future studies should prioritize sufficiently large sample sizes to enable robust evaluation and effective bias mitigation.^70^

Data privacy is critical in pediatric LLM research. Although methods exist to deidentify large clinical datasets, they are not fully reliable and residual risk of reidentification remains.^71^ Many studies relied solely on deidentification to address privacy concerns, and few (6 of 40) reported using HIPAA-compliant models. The frequent use of publicly hosted GPT tools (eg, ChatGPT), which do not operate in HIPAA-compliant environments, further elevates the risk to sensitive patient data.^72^ Future research should prioritize HIPAA-compliant or locally deployed models and explicitly outline robust privacy safeguards beyond deidentification.

Reporting limitations were evident across studies. Although basic study characteristics, such as population, setting, and data source, were generally reported, few studies fully adhered to MINIMAR standards, with most missing critical details, such as patient demographics and data missingness. The lack of demographic and socioeconomic reporting may contribute to unrecognized bias and limits equity assessment in pediatric LLM research. Although MINIMAR elements of model optimization and internal validation were assessed only in studies that trained or fine-tuned an LLM, adherence to these reporting elements varied across studies. Only 1 study met all MINIMAR criteria,^19^ highlighting persistent gaps in transparency and reproducibility. These findings underscore the need for standardized reporting frameworks, such as MINIMAR^19^ or TRIPOD-LLM,^73^ as well as pediatric-specific guidance, such as ACCEPT-AI,^74^ to support comprehensive, consistent, and equity-focused reporting standards in pediatric LLM research.

Lastly, no studies engaged external stakeholders in study design or interpretation. Future research should meaningfully involve key stakeholders, including parents and caregivers, throughout model development to ensure patient-centered, contextually appropriate LLM implementation.^75^

### Strengths and Limitations

This scoping review has both strengths and limitations. Strengths of this review include a comprehensive search across multiple databases and preprint servers, a robust search strategy targeting pediatric and LLM-relevant studies, and inclusion of studies using clinical rather than synthetic or theoretical data. Limitations include the inclusion of non–peer-reviewed preprints and conference proceedings due to the rapidly evolving nature of LLM research; restriction to English-language publications; possible omission of studies published after the search dates; exclusion of multimodal LLM systems; and the use of publication-type filters that may be inconsistently applied in electronic databases, potentially leading to inadvertent exclusion of relevant studies. In addition, publication bias favoring positive or statistically significant findings may have influenced the available literature. Additionally, this review was limited to studies explicitly focused on pediatric populations; consequently, LLM use cases more common in mixed-age or general populations, such as clinical documentation and text summarization, may be underrepresented. Despite these limitations, this scoping review provides important conclusions to inform future research on AI applications in pediatrics.

## Conclusions

This scoping review mapped the emerging literature on LLM applications in pediatric clinical text, highlighting promising use cases and key limitations. As LLMs enter pediatric workflows, it is crucial to consider the unique aspects of pediatric care. Future research should prioritize rigorous study designs, pediatric-specific models, underrepresented specialties and age groups, and stakeholder input, while adhering to implementation, evaluation, and reporting standards to support safe, effective, and equitable deployment of LLMs in pediatrics.

## Notes

Supplement 1. **eTable 1.** Full Search Strategy **eTable 2.** Data Extraction for Scoping Review **eTable 3.** Characteristics of Included Studies, Grouped by Broader Pediatric Medical Specialty Categories

Supplement 2.
Data Sharing Statement
