# Supplementary material for: Large Language Models Using Clinical Text in Pediatrics: A Scoping Review
Source: JAMA Netw Open. 2026 Mar 25;9(3):e262443. doi: 10.1001/jamanetworkopen.2026.2443 (PMC13019234; doi:10.1001/jamanetworkopen.2026.2443)
Supplement: Supplement 1. — eTable 1. Full Search Strategy eTable 2. Data Extraction for Scoping Review eTable 3. Characteristics of Included Studies, Grouped by Broader Pediatric Medical Specialty Categories [file jamanetwopen-e262443-s001.pdf]

Supplemental Online Content

Huang T, Tse G, Pageler NM, Bannett Y. Large language models using clinical text in pediatrics. *JAMA Netw Open*. 2026;9(3):e262443.
doi:10.1001/jamanetworkopen.2026.2443

eTable 1. Full Search Strategy

eTable 2. Data Extraction for Scoping Review

eTable 3. Characteristics of Included Studies, Grouped by Broader Pediatric Medical Specialty Categories

This supplemental material has been provided by the authors to give readers additional information about their work.

© 2026 Huang T et al. *JAMA Network Open*.

eTable 1. Full Search Strategy

Database	Search Query	# of Search Results	Notes/ Field Tags	Publication Type Filters
PubMed	(infan*[tiab] OR newborn*[tiab] OR "new-born"[tiab] OR "new born*"[tiab] OR perinat*[tiab] OR neonat*[tiab] OR baby*[tiab] OR babies[tiab] OR toddler*[tiab] OR minors[tiab] OR child*[tiab] OR children*[tiab] OR adolescen*[tiab] OR juvenil*[tiab] OR youth*[tiab] OR teen*[tiab] OR "under age"[tiab] OR pubescen*[tiab] OR prepubesc*[tiab] OR "puberty"[tiab] OR pediatric*[tiab] OR paediatric*[tiab] OR "infant"[mesh] OR "Child"[mesh] OR "Adolescent"[mesh] OR "Minors"[mesh] OR "Puberty"[mesh] OR "Pediatrics"[mesh])<br><br>AND ("Large Language Models"[mesh] OR "Generative language model*"[tiab] OR "Mistral"[tiab] OR "Mixtral"[tiab] OR "Bloomz"[tiab] OR "Qwen"[tiab] OR "Gemma"[tiab] OR "bard"[tiab] OR "grok"[tiab] OR "meta ai"[tiab] OR "copilot"[tiab] OR "perplexity-ai"[tiab] OR "open ai"[tiab] OR "azure"[tiab] OR "claude"[tiab] OR "gemini"[tiab] OR "sonnet"[tiab] OR "llama"[tiab] OR "flan"[tiab] OR "pathways language model"[tiab] OR "transformer model*"[tiab] OR "foundation model*"[tiab] OR "large language model*"[tiab] OR "deepseek"[tiab] OR chatgpt[tiab] OR "chat gpt"[tiab] OR "chat-gpt"[tiab] OR "gpt"[tiab] OR "generative pre-trained"[tiab] OR "generative ai"[tiab] OR "generative artificial"[tiab] OR "pre trained language model*"[tiab] OR llm[tiab] OR llms[tiab])<br><br>AND 2020:3000 [dp]<br><br>AND English [lang]	153	Title, Abstract	- Adaptive Clinical Trial<br>- Case Reports<br>- Classical Article<br>- Clinical Study<br>- Clinical Trial<br>- Clinical Trial, Phase I<br>- Clinical Trial, Phase II<br>- Clinical Trial, Phase III<br>- Clinical Trial, Phase IV<br>- Comparative Study<br>- Controlled Clinical Trial<br>- Corrected and Republished Article<br>- Equivalence Trial<br>- Evaluation Study<br>- Letter<br>- Multicenter Study<br>- Observational Study<br>- Pragmatic Clinical Trial<br>- Preprint<br>- Randomized Controlled Trial<br>- Validation Study

© 2026 Huang T et al. *JAMA Network Open*.

**Database**	**Search Query**	**# of Search Results**	**Notes/ Field Tags**	**Publication Type Filters**
Embase (Embase, Preprints, Clinical Trials)	('infan*':ti,ab,kw OR 'newborn*':ti,ab,kw OR 'new-born':ti,ab,kw OR 'new born*':ti,ab,kw OR 'perinat*':ti,ab,kw OR 'neonat*':ti,ab,kw OR 'baby*':ti,ab,kw OR 'babies':ti,ab,kw OR 'toddler*':ti,ab,kw OR 'minors':ti,ab,kw OR 'child*':ti,ab,kw OR 'children*':ti,ab,kw OR 'adolescen*':ti,ab,kw OR 'juvenil*':ti,ab,kw OR 'youth*':ti,ab,kw OR 'teen*':ti,ab,kw OR 'under age':ti,ab,kw OR 'pubescen*':ti,ab,kw OR 'prepubesc*':ti,ab,kw OR 'puberty':ti,ab,kw OR 'pediatric*':ti,ab,kw OR 'paediatric*':ti,ab,kw OR 'infant'/exp OR 'child'/exp OR 'adolescent'/exp OR 'minor (person)'/exp OR 'puberty'/exp OR 'pediatrics'/exp)<br><br>AND ('large language model'/exp OR 'generative language model*':ti,ab,kw OR 'mistral':ti,ab,kw OR 'mixtral':ti,ab,kw OR 'bloomz':ti,ab,kw OR 'qwen':ti,ab,kw OR 'gemma':ti,ab,kw OR 'bard':ti,ab,kw OR 'grok':ti,ab,kw OR 'meta ai':ti,ab,kw OR 'copilot':ti,ab,kw OR 'perplexity-ai':ti,ab,kw OR 'open ai':ti,ab,kw OR 'azure':ti,ab,kw OR 'claude':ti,ab,kw OR 'gemini':ti,ab,kw OR 'sonnet':ti,ab,kw OR 'llama':ti,ab,kw OR 'flan':ti,ab,kw OR 'pathways language model':ti,ab,kw OR 'transformer model*':ti,ab,kw OR 'foundation model*':ti,ab,kw OR 'large language model*':ti,ab,kw OR 'deepseek':ti,ab,kw OR 'chatgpt':ti,ab,kw OR 'chat gpt':ti,ab,kw OR 'chat-gpt':ti,ab,kw OR 'gpt':ti,ab,kw OR 'generative pre-trained':ti,ab,kw OR 'generative ai':ti,ab,kw OR 'generative artificial':ti,ab,kw OR 'pre trained language model*':ti,ab,kw OR 'llm*':ti,ab,kw)<br><br>AND ('article'/it OR 'article in press'/it OR 'conference paper'/it OR 'letter'/it OR 'preprint'/it OR 'clinical trial'/it)<br><br>AND [english]/lim<br><br>AND [2020-2025]/py	929	Title, Abstract	- Article<br>- Article in Press<br>- Conference Paper<br>- Letter<br>- Preprint<br>- Clinical Trial

© 2026 Huang T et al. *JAMA Network Open*.

**Database**	**Search Query**	**# of Search Results**	**Notes/ Field Tags**	**Publication Type Filters**
Scopus	(TITLE-ABS (infan* OR newborn* OR "new-born" OR "new born*" OR perinat* OR neonat* OR baby* OR babies OR toddler* OR minors OR child* OR children* OR adolescen* OR juvenil* OR youth* OR teen* OR "under age" OR “puberty” OR pubescen* OR prepubesc* OR pediatric* OR paediatric*))<br><br>AND (TITLE-ABS("large language model*" OR "generative language model*" OR Mistral OR Mixtral OR Bloomz OR Qwen OR Gemma OR bard OR grok OR "meta ai" OR copilot OR "perplexity-ai" OR “open ai” OR azure OR claude OR gemini OR sonnet OR llama OR flan OR "pathways language model" OR "transformer model*" OR "foundation model*" OR deepseek OR chatgpt OR "chat gpt" OR “chat-gpt” OR gpt OR "generative pre-trained" OR "generative ai" OR "generative artificial" OR "pre trained language model*" OR llm*))<br><br>AND LIMIT-TO(LANGUAGE, "English")<br><br>AND PUBYEAR > 2019 AND PUBYEAR < 2026	1,710	Title, Abstract	- Article<br>- Conference Paper<br>- Letter

© 2026 Huang T et al. *JAMA Network Open*.

**Database**	**Search Query**	**# of Search Results**	**Notes/ Field Tags**	**Publication Type Filters**
Web of Science	(TI=(infan* OR newborn* OR "new-born" OR "new born*" OR perinat* OR neonat* OR baby* OR babies OR toddler* OR minors OR child* OR children* OR adolescen* OR juvenil* OR youth* OR teen* OR "under age" OR “puberty” OR pubescen* OR prepubesc* OR pediatric* OR paediatric*)<br><br>OR AB=(infan* OR newborn* OR "new-born" OR "new born*" OR perinat* OR neonat* OR baby* OR babies OR toddler* OR minors OR child* OR children* OR adolescen* OR juvenil* OR youth* OR teen* OR "under age" OR “puberty” OR pubescen* OR prepubesc* OR pediatric* OR paediatric*))<br><br>AND<br><br>(TI=("large language model*" OR "generative language model*" OR Mistral OR Mixtral OR Bloomz OR Qwen OR Gemma OR bard OR grok OR "meta ai" OR copilot* OR "perplexity-ai" OR “open ai” OR azure OR claude OR gemini OR sonnet OR llama OR flan OR "pathways language model” OR "transformer model*" OR "foundation model*" OR deepseek OR chatgpt OR "chat gpt" OR “chat-gpt” OR gpt OR "generative pre-trained" OR "generative ai" OR "generative artificial" OR "pre trained language model*" OR llm*)<br><br>OR AB=("large language model*" OR "generative language model*" OR Mistral OR Mixtral OR Bloomz OR Qwen OR Gemma OR bard OR grok OR "meta ai" OR copilot* OR "perplexity-ai" OR “open ai” OR azure OR claude OR gemini OR sonnet OR llama OR flan OR "pathways language model” OR "transformer model*" OR "foundation model*" OR deepseek OR chatgpt OR "chat gpt" OR “chat-gpt” OR gpt OR "generative pre-trained" OR "generative ai" OR "generative artificial" OR "pre trained language model*" OR llm*))<br><br>AND LA=English<br><br>AND PY=(2020-2025)	1,293	Title, Abstract	- Article<br>- Proceeding Paper<br>- Letter<br>- Early Access

**Database**	**Search Query**	**# of Search Results**	**Notes/ Field Tags**	**Publication Type Filters**
arXiv	N/A; Identified from Scopus search and then manually screened for relevance	23	N/A	N/A
medRxiv	N/A; Identified from Scopus search and then manually screened for relevance	2	N/A	N/A
Research Square	N/A; Identified from Scopus search and then manually screened for relevance	3	N/A	N/A
Manually Identified Article	N/A	1	N/A	N/A

© 2026 Huang T et al. *JAMA Network Open*.

© 2026 Huang T et al. *JAMA Network Open*.

eTable 2. Data Extraction for Scoping Review

**Domain**	**Information Extracted**
Publication and Study Characteristics	• Study Name<br>• Study Location<br>• Year of Publication<br>• Study Design<br>• Publication Type<br>• Study Dates<br>• Study Duration<br>• Pediatric Data as Primary or Secondary Data Source
Pediatric Population Characteristics	• Age-Related Statistics<br>• Age Range<br>• Age Subgroups Represented<br>• Population Description<br>• Population Sample Size<br>• Cohort Selection<br>• Sex<br>• Race<br>• Ethnicity<br>• Socioeconomic Status
Clinical Data Input Characteristics	• Input Data Description<br>• Input Data Type<br>• Input Language<br>• Data Source<br>• Data Collection Mode<br>• Missingness
Clinical Context Characteristics	• Clinical Setting<br>• Clinical Subspecialty<br>• Clinical Condition
LLM Characteristics	• LLM Used<br>• LLM Developer<br>• Number of LLMs Evaluated
LLM Implementation Characteristics	• LLM Use Case/Task<br>• Model Output<br>• Target User<br>• Data Splitting<br>• Model Architecture<br>• Input Features<br>• Model Optimization<br>• Training/Validation Sample Size<br>• Evaluation Sample Size<br>• Pre-Trained or Fine-Tuned LLM

© 2026 Huang T et al. *JAMA Network Open*.

Domain	Information Extracted
LLM Evaluation and Validation Characteristics	• Evaluation Methods<br>• Performance Metrics<br>• Annotation Method, # of Annotators, and IAA Reported<br>• Gold Standard<br>• Comparator<br>• Internal Validation<br>• External Validation
LLM Outcome Characteristics	• Outcome Summary<br>• Identified Benefits<br>• Identified Limitations
Ethical, Regulatory, and Risk Considerations	• Discussion of Ethics/Data Privacy<br>• Use of HIPAA-Compliant LLM<br>• Fairness Analysis<br>• Subgroup Analysis<br>• Error Analysis<br>• Bias Mitigation Efforts<br>• Stakeholders Involved<br>Data and Code Sharing Transparency

© 2026 Huang T et al. *JAMA Network Open*.

eTable 3. Characteristics of Included Studies, Grouped by Broader Pediatric Medical Specialty Categories

Pediatrics Subspecialty	Population	Pediatrics-Specific Challenge	LLM Use Case	LLM Use Case Category / Sub-Category / Task*	LLM Name and Developer	Data Source	Sample Size	Publication
**Pediatric Medical Specialties**								
Fetal Cardiology	Pregnant women who had fetal echocardiography	Timely referral for fetal echocardiography is critical for early diagnosis of congenital heart defects, a leading cause of infant morbidity and mortality	Evaluate indication for fetal echocardiography based on American Institute of Ultrasound in Medicine (AIUM) guidelines	Clinical Decision Support<br>• Diagnostic Decision Support<br>○ Recognize disease patterns from symptoms/vitals	GPT-4 (OpenAI)	Clinical notes from Shamir Medical Center	59 clinical notes	Kopylov et al,[33] 2024
Pediatric Clinical Genetics	Patients with clinical notes, manually selected for their clear disease/gene labels and rich patient information	Rare genetic diseases are more common in pediatric patients and are difficult to diagnose due to complex, variable phenotypes and limited structured data	Conduct rare genetic disorders diagnosis and gene prioritization	Clinical Decision Support<br>• Diagnostic Decision Support<br>○ Recognize disease patterns from symptoms/vitals	LLaMA 3.3-70B-Instruct (Meta AI), DeepSeek-R1-Distill- LLaMA-70B (DeepSeek)	EHR clinical notes from Children's Hospital of Philadelphia	220 clinical notes	Wu et al,[52] 2025
Pediatric Infectious Diseases	Children seen in the emergency department with severe illness	COVID-19 severity in children is highly variable and may not follow adult patterns, complicating early outcome prediction and risk stratification[a]	Classify patient outcomes as severe or non-severe COVID-19	Clinical Decision Support<br>• Predicting Risks and Outcomes<br>○ Predict outcomes, adverse events, discharge readiness	LLaMA 2–7B (Meta AI), Flan-T5-XL (Google), Flan-T5-XXL (Google), T0pp (BigScience), T0-3B (BigScience)	Participant survey records, including demographics and health status from Children's Hospital of Michigan and UPMC Children's	383 records[b]	Roshani et al,[45] 2025

						Hospital of Pittsburgh		
**Pediatrics Subspecialty**	**Population**	**Pediatrics-Specific Challenge**	**LLM Use Case**	**LLM Use Case Category / Sub-Category / Task***	**LLM Name and Developer**	**Data Source**	**Sample Size**	**Publication**
Pediatric Infectious Diseases	Patients with a primary diagnosis of pediatric pneumonia	Similar symptoms across pediatric respiratory diseases complicate accurate diagnosis of pediatric pneumonia, leading to misdiagnosis and inappropriate treatment	Simulate and generate structured clinical documentation for pneumonia outpatient visits, hospitalization, and physician ward rounds, including diagnosis, treatment plans, and clinical reasoning	Clinical Note Generation<br>• Documenting Care Plans<br>○ Document treatment plans, care protocols, nursing plans, advance planning<br><br>Clinical Decision Support<br>• Diagnostic Decision Support<br>○ Generate differential diagnoses<br><br>Clinical Decision Support<br>• Treatment Planning<br>○ Suggest clinical pathways	Custom LLM called P2Med-MLLM, Baichuan 2 (Baichuan AI) and Chinese-LLaMA 2-7B (Meta AI)	EHR radiology, outpatient, and inpatient records from Children's Hospital of Fudan University	400 records[b]	Tian et al,[47] 2025
Pediatric Infectious Diseases	Patients who had visits with either an ICD-10-CM code for acute sinusitis or chronic sinusitis and a prescription of an oral antibiotic	Pediatric antibiotic prescriptions for sinusitis are often inappropriate due to overlapping viral symptoms and lack of structured data, requiring time-intensive manual chart reviews to assess prescribing appropriateness	Classify the appropriateness of antibiotic prescriptions for sinusitis	Clinical Decision Support<br>• Treatment Planning<br>○ Match protocols/screen contraindications	LLaMA 3-70B-Instruct (Meta AI), LLaMA 3.1-405B-Instruct (Meta AI)	Clinical encounter notes from Children's Hospital of Philadelphia network	50 clinical notes[b]	Weissenbacher et al,[51] 2025

© 2026 Huang T et al. *JAMA Network Open*.

**Pediatrics Subspecialty**	**Population**	**Pediatrics-Specific Challenge**	**LLM Use Case**	**LLM Use Case Category / Sub-Category / Task***	**LLM Name and Developer**	**Data Source**	**Sample Size**	**Publication**
Pediatric Nephrology	Patients who were followed up with a diagnosis of acute post-streptococcal glomerulonephritis (APSGN) in the department of pediatric nephrology	Acute post-streptococcal glomerulonephritis (remains a significant pediatric kidney disease in low-resource settings, requiring timely diagnosis and complex management to prevent complications	Provide diagnosis and treatment recommendations for APSGN	Clinical Decision Support<br>• Diagnostic Decision Support<br>○ Generate differential diagnoses<br><br>Clinical Decision Support<br>• Treatment Planning<br>○ Suggest clinical pathways	GPT-3.5 (OpenAI)	Clinical cases including clinical, lab, and biopsy data from Clinic of Pediatric Nephrology at Konya City Hospital	11 cases	Leventoglu et al,[35] 2024
Pediatric Nephrology	Patients undergoing cardiopulmonary bypass surgery	Clinical notes for pediatric cardiovascular bypass surgery patients lack structured labeling, limiting early acute kidney injury prediction and delaying intervention	Label clusters of patients grouped by operation and diagnosis	Clinical Decision Support<br>• Predicting Risks and Outcomes<br>○ Predict outcomes, adverse events, discharge readiness	Gemini-1.5-Pro (Google) and GPT-4o (OpenAI)	EHR clinical records, including demographic information, surgical details, and standardized diagnosis/procedure codes from Great Ormond Street Hospital	963 records	Sharabiani et al,[38] 2025

© 2026 Huang T et al. *JAMA Network Open*.

**Pediatrics Subspecialty**	**Population**	**Pediatrics-Specific Challenge**	**LLM Use Case**	**LLM Use Case Category / Sub-Category / Task**[*]	**LLM Name and Developer**	**Data Source**	**Sample Size**	**Publication**
Pediatric Neurology	Patients with ischemic stroke or cerebral venous sinus thrombosis	Data entry for large registries like the International Pediatric Stroke Study (IPSS) is time-consuming and burdensome for investigators	Extract and populate structured data for the IPSS registry	Medical Research Assistance<br>• Analyzing Research Data<br>○ Conduct cohort studies	GPT-3.5 (OpenAI)	EHR clinical notes from UTHealth Pediatric Stroke Clinic	50 clinical notes	Fiedler et al,[23] 2024
Pediatric Oncology	Pediatric patients with osteosarcoma	Pathology reports for pediatric osteosarcoma are often complex and unstructured, making it challenging to extract critical details like tumor grade and margin status for treatment planning and outcome prediction[a]	Extract pathological classifications (histology grade and margin status)	Clinical Note Generation<br>• Documenting Diagnostic Reports<br>○ Generate imaging, pathology, test, and genomic reports	GPT-3.5-Turbo-16K (OpenAI)	Pathology reports from UT Southwestern Medical Center	191 reports[b]	Huang et al,[25] 2024
Pediatric Oncology	Pediatric patients who underwent genetic testing and had pathogenic CpG mutations	Clinical notes often lack structured identification of cancer predisposition syndromes in children, limiting timely diagnosis and appropriate surveillance	Recommend cancer predisposition syndromes (CPS) genes	Clinical Decision Support<br>• Diagnostic Decision Support<br>○ Recognize disease patterns from symptoms/vitals	GPT-3.5 (OpenAI)	Clinical notes from first visits and genetic consultations at King Hussein Cancer Center (KHCC)	53 notes	Sultan et al,[46] 2023

© 2026 Huang T et al. *JAMA Network Open*.

**Pediatrics Subspecialty**	**Population**	**Pediatrics-Specific Challenge**	**LLM Use Case**	**LLM Use Case Category / Sub-Category / Task***	**LLM Name and Developer**	**Data Source**	**Sample Size**	**Publication**
**Pediatric Surgical Specialties**								
General Pediatric Surgery	Children who underwent surgery for perforated appendicitis confirmed intraoperatively	Key clinical variables for pediatric appendicitis severity are buried in unstructured operative/ultrasound reports, limiting efficient complication grading and standardization	Extract structured clinical variables and estimate appendicitis severity grade	Clinical Decision Support<br>• Diagnostic Decision Support<br>○ Interpret diagnostic tests<br><br>Clinical Decision Support<br>• Predicting Risks and Outcomes<br>○ Predict deterioration, readmission, disease progression	GPT-4 (OpenAI)	Ultrasound and operative reports from Montreal Children Hospital	103 reports	Abu-Ashour et al,[14] 2024
Pediatric Cardiovascular Surgery	Patients who underwent cardiovascular surgery	There is a lack of reliable, accessible guidance on diagnosis, surgical approach, and timing of surgery for congenital heart disease in pediatric cardiac surgery	Summarize cardiovascular surgery cases, identify diagnoses, and recommend surgical procedures and timing	Clinical Note Generation<br>• Documenting Patient Visits<br>○ Summarize clinical documents<br><br>Clinical Decision Support<br>• Diagnostic Decision Support<br>○ Generate differential diagnoses<br><br>Clinical Decision Support<br>• Treatment Planning	GPT-4 (OpenAI)	Clinical cases, including cardiac imaging and monitoring data, progress notes, and patient summaries from a cardiovascular surgery conference at a single tertiary center	37 cases	Mehta et al,[42] 2025

© 2026 Huang T et al. *JAMA Network Open*.

				○ Suggest clinical pathways				
**Pediatrics Subspecialty**	**Population**	**Pediatrics-Specific Challenge**	**LLM Use Case**	**LLM Use Case Category / Sub-Category / Task***	**LLM Name and Developer**	**Data Source**	**Sample Size**	**Publication**
Pediatric Ophthalmology	Pediatric outpatients with myopia, hyperopia, and emmetropia	Rising prevalence of pediatric refractive errors requires early intervention, but expert evaluations are resource-intensive and limited in low-access settings	Determine whether intervention is needed and recommend treatment	Clinical Decision Support<br>• Treatment Planning<br>○ Suggest clinical pathways	GPT-3.5 (OpenAI), GPT-4o (OpenAI), Wenxin Yiyan (Baidu)	Refractive error cases from Children's Hospital, Zhejiang University School of Medicine	100 cases	Kang et al,[31] 2025
Pediatric Orthopedics	Patients with previously diagnosed single curve scoliosis	Radiologic documentation of scoliosis in pediatric patients is often unstructured, making it difficult to extract consistent curve classification for diagnosis and treatment planning[a]	Classify single-curve scoliosis	Clinical Decision Support<br>• Diagnostic Decision Support<br>○ Recognize disease patterns from symptoms/vitals	GPT-4 (OpenAI), Microsoft Bing (Microsoft), Scholar AI (Scholar AI)	Radiologic descriptions of single-curve scoliosis cases from Polish-Mother's Memorial Hospital Research Institute	56 cases	Fabijan et al,[21] 2023
Pediatric Otolaryngology	Pediatric patients with a confirmed diagnosis of sialadenitis	Pediatric sialadenitis presents complex, age-specific management challenges with unclear indications for sialendoscopy	Provide differential diagnoses and suggest further tests and treatments	Clinical Decision Support<br>• Diagnostic Decision Support<br>○ Generate differential diagnoses<br><br>Clinical Decision Support<br>• Treatment Planning<br>○ Suggest clinical pathways	GPT-4 (OpenAI)	Clinical cases, including clinical notes, imaging reports, and laboratory results from San Sebastian University Hospital	49 cases	Maniaci et al,[39] 2024

© 2026 Huang T et al. *JAMA Network Open*.

Pediatric Plastic Surgery	Patients who underwent craniofacial surgery	Craniofacial procedures in children are complex and highly individualized, and operative notes often lack standardized language, making accurate CPT coding difficult and error-prone[a]	Provide appropriate CPT codes	Administration and Workflow<br>• Overseeing Financial Activities<br>○ Generate/document billing, insurer communication	Gemini (Google), GPT-4 (OpenAI)	Craniofacial surgery operative notes from Nemours Children's Health	10 operative notes	Isch et al,[28] 2025
**Pediatrics Subspecialty**	**Population**	**Pediatrics-Specific Challenge**	**LLM Use Case**	**LLM Use Case Category / Sub-Category / Task***	**LLM Name and Developer**	**Data Source**	**Sample Size**	**Publication**
**Pediatric Emergency and Critical Care Medicine**								
Pediatric Critical Care Medicine	Pediatric patients admitted to the pediatric intensive care unit (PICU)	Critically ill children often present with non-specific or overlapping symptoms, making differential diagnosis in the PICU especially complex and time-sensitive[a]	Generate differential diagnoses for PICU patients	Clinical Decision Support<br>• Diagnostic Decision Support<br>○ Generate differential diagnoses	BioGPT-Large (Microsoft) , LLaMA 65B (Meta AI), LLaMA 2-7B (Meta AI)	Clinical notes, including admission, progress, discharge, procedure, and consultant notes, from Boston Children's Hospital	130 clinical notes[b]	Akhondi-Asl et al,[16] 2024
Pediatric Emergency Medicine	Pediatric patients with emergency room admissions	Pediatric emergency care often has poorly labeled patient complaints in triage notes, causing delays in treatment	Classify if notes align with characteristics of a respiratory tract infection (RTI)	Clinical Decision Support<br>• Diagnostic Decision Support<br>○ Recognize disease patterns from symptoms/vitals	text-davinci-003" model (OpenAI)	Initial triage assessment notes from Hacettepe University İhsan Doğramacı Children's Hospital	5350 assessment notes	Akbasli et al,[15] 2025
Pediatric Emergency Medicine	Emergency medical service-	Abusive head trauma and child abuse detection in young	Detect abusive head	Clinical Decision Support<br>• Diagnostic Decision Support	GPT-4o (OpenAI)	Emergency medical service free-	1082 narratives	Broad et al,[20] 2025

© 2026 Huang T et al. *JAMA Network Open*.

	transported patients with at least one CDC-recommended ICD-10 diagnosis for abusive head trauma	children is critical but challenging for EMS clinicians	trauma-child abuse and neglect	○ Recognize disease patterns from symptoms/vitals		text narratives ESO Data Collaborative		
**Pediatrics Subspecialty**	**Population**	**Pediatrics-Specific Challenge**	**LLM Use Case**	**LLM Use Case Category / Sub-Category / Task***	**LLM Name and Developer**	**Data Source**	**Sample Size**	**Publication**
Pediatric Emergency Medicine	Patient records with free-text discharge diagnoses manually classified as unintentional injuries per WHO injury classification system	Pediatric emergency department (ED) injury data is often located in free text, preventing timely and accurate identification of injury data	Identify and classify different types of injuries	Clinical Decision Support<br>• Diagnostic Decision Support<br>○ Recognize disease patterns from symptoms/vitals	GPT-4 (OpenAI)	EHR free-text records from Padova University Hospital	8194 records	Lorenzoni et al,[37] 2024
Pediatric Emergency Medicine	Patients with psychiatric emergency department visits	Psychiatric symptoms in children and adolescents often present atypically in emergency settings, making accurate assessment of psychopathology dimensions difficult under time constraints[a]	Estimate dimensional psychopathology	Clinical Decision Support<br>• Diagnostic Decision Support<br>○ Recognize disease patterns from symptoms/vitals	gpt-4-1106-preview (OpenAI)	Emergency psychiatry clinical notes from unspecified psychiatric emergency department of a large academic medical center	3059 clinical notes	McCoy and Perlis,[41] 2024
Neonatology	Infants who obtained a radiograph while admitted to a large neonatal	Neonatal radiology reports are typically unstructured and contain subtle findings, making it difficult to consistently identify	Extract disease-status labels	Clinical Note Generation<br>• Documenting Diagnostic Reports<br>○ Generate imaging, pathology, test, and	Mixtral-8x7B (Mistral)	Radiology reports from Beth Israel Deaconess Medical Center	15,795 reports	Huang et al,[26] 2025

© 2026 Huang T et al. *JAMA Network Open*.

	intensive care unit	disease status for timely diagnosis and intervention[a]		genomic reports				
Neonatology	Neonates	Neonatal clinical data is often embedded in unstructured narratives, making it difficult to retrieve key information for diagnosis, monitoring, and care coordination[a]	Extract and structure medical information	Clinical Note Generation<br>• Documenting Patient Visits<br>○ Summarize clinical documents	GPT-3.5 (OpenAI), GPT-4 (OpenAI)	Free-text neonatal medical reports from Mohammed VI University Hospital	100 reports	Kaddari et al,[30] 2024
**Pediatrics Subspecialty**	**Population**	**Pediatrics-Specific Challenge**	**LLM Use Case**	**LLM Use Case Category / Sub-Category / Task[*]**	**LLM Name and Developer**	**Data Source**	**Sample Size**	**Publication**
Neonatology	Patients who received an abdominal X-ray in the neonatal intensive care unit (NICU)	Early detection of neonatal necrotizing enterocolitis (NEC) is critical but hampered by the lack of automated, expert-level labeling of abdominal X-ray reports, limiting timely diagnosis and treatment	Detect necrotizing enterocolitis and classify its subtypes	Clinical Decision Support<br>• Diagnostic Decision Support<br>○ Recognize disease patterns from symptoms/vitals	Gemma-2B-it (EleutherAI), Gemma-7B-it (EleutherAI), Mistral-7B-it (Mistral AI)	Abdominal X-ray reports from C.S. Mott Children's Hospital	437 reports[b]	Zhang et al,[53] 2025
**Developmental, Behavioral, and Mental Health**								
Child and Adolescent Psychiatry	Pediatric patients with a diagnosis of depression or related mood disorders	Depression in pediatric populations is frequently under-detected in primary care due to challenges in screening from unstructured clinical text, inconsistent interpretation of symptoms, and limited use of	Extract relevant depression-related symptoms	Clinical Decision Support<br>• Diagnostic Decision Support<br>○ Recognize disease patterns from symptoms/vitals	FLAN T5 (Google Research), LLaMA 3-70B (Meta AI), Phi 3.5 mini (Microsoft Research)	Clinical notes from Cincinnati Children's Hospital Medical Center	85 clinical notes	Ignashina et al,[27] 2025

© 2026 Huang T et al. *JAMA Network Open*.

		standardized tools like the PHQ-9						
Developmental-Behavioral Pediatrics	Children with ADHD and ≥2 encounters, including visits with ADHD diagnosis or prescription of stimulants or nonstimulants	Lack of scalable methods to capture attention deficit hyperactivity disorder (ADHD) medication management from unstructured clinical notes limits accurate assessment of guideline adherence in pediatric care	Classify ADHD medication side effect inquiry	Clinical Decision Support<br>• Providing Clinical Knowledge Support<br>○ Assess care quality	LLaMA 2-13B (Meta AI)	Clinical notes from Packard Children's Health Alliance (PCHA)	363 clinical notes[b]	Bannett et al,[19] 2025
**Pediatrics Subspecialty**	**Population**	**Pediatrics-Specific Challenge**	**LLM Use Case**	**LLM Use Case Category / Sub-Category / Task***	**LLM Name and Developer**	**Data Source**	**Sample Size**	**Publication**
Developmental-Behavioral Pediatrics	Verbally fluent children with and without ASD symptoms	Child-clinician conversations during autism assessments are complex and unstructured, limiting clinicians' ability to efficiently analyze behaviors and language skills critical for accurate autism diagnosis	Classify speakers, recognize activities and emotional cues, assess language skills, and predict developmental traits	Clinical Decision Support<br>• Diagnostic Decision Support<br>○ Recognize disease patterns from symptoms/vitals	Mistral-7B V0.2 (Mistral), LLaMa 2-7B (Meta AI), LLaMa 2-13B (Meta AI), LLaMa 3-8B- Instruct-2 (Meta AI), Qwen1.5-7B (Alibaba Cloud), Qwen1.5-14B (Alibaba Cloud)	Autism child-clinician interaction transcripts from the National Institute of Mental Health Data Archive	164 transcripts	Feng et al,[22] 2024
Developmental-Behavioral Pediatrics	Patients with suspected autism	Autism diagnoses in children rely on expert interpretation of complex clinical interactions, but limited specialist availability and variability in behavior lead to missed or delayed diagnoses	Generate scores and justifications for the eight items used in the ADOS clinical diagnoses	Clinical Decision Support<br>• Diagnostic Decision Support<br>○ Recognize disease patterns from symptoms/vitals	Yi-34b (01.AI), glm4 (THUDM / Zhipu AI), glm-3-turbo (THUDM / Zhipu AI), kimi (Moonshot AI), Qwen-max (Alibaba / Qwen), Qwen1.5-110b (Alibaba / Qwen), Qwen1.5-72b	Autism assessment process transcripts from unspecified outpatient clinic	28 transcripts	Jiang et al,[29] 2024

© 2026 Huang T et al. *JAMA Network Open*.

					(Alibaba / Qwen), Qwen1.5-32b (Alibaba / Qwen), Qwen1.5-14b (Alibaba / Qwen), GPT-4 (OpenAI), Gemini-1.5-Pro (Google DeepMind), Claude-3-Opus (Anthropic), Mixtral-8x22b (Mistral), Mixtral-8x7b (Mistral), Mistral-7b (Mistral), LLaMA 3-8b (Meta AI)			
**Pediatrics Subspecialty**	**Population**	**Pediatrics-Specific Challenge**	**LLM Use Case**	**LLM Use Case Category / Sub-Category / Task***	**LLM Name and Developer**	**Data Source**	**Sample Size**	**Publication**
Developmental-Behavioral Pediatrics	Children with rare genetic neurodevelopmental disorder and children with cerebral palsy	Standard assessments of verbal and ambulatory ability in children with developmental disabilities are burdensome and inconsistent, limiting longitudinal monitoring of functional status	Categorize if the patient can use any words or walk without aid	Clinical Decision Support<br>• Diagnostic Decision Support<br>○ Recognize disease patterns from symptoms/vitals	GPT-3.5 Turbo (Open AI), GPT-4 Turbo (Open AI), GPT-4o (Open AI)	Clinical notes from National Brain Gene Registry and Washington University School of Medicine's Research Data Core	3,245 notes (first dataset) and 5,462 clinical notes (second dataset)	Kaster et al,[32] 2025
Developmental-Behavioral Pediatrics	Patients diagnosed with autism spectrum disorder	The growing demand for individualized Applied Behavior Analysis (ABA) therapy in children with autism is hindered by a shortage of trained	Generate personalized treatment plans for ABA	Clinical Decision Support<br>• Treatment Planning<br>○ Suggest clinical pathways	Flan-T5-base (Google Research), Flan-T5-large (Google Research), Phi-2b (Microsoft Research), custom LLM	Patient cases, including raw assessment files, diagnostic reports, and parent interview	123 patient cases	Kumar et al,[34] 2024

© 2026 Huang T et al. *JAMA Network Open*.

		clinicians, delaying timely and tailored treatment planning			based on Flan-T5-base	forms from unspecified outpatient clinic		
Developmental-Behavioral Pediatrics	Children aged 4 - 42 months	Developmental screening in young children, especially preterm infants, is often time- and resource-intensive, limiting timely assessment and intervention	Conduct pediatric developmental screening for developmental delays	Clinical Decision Support<br>• Predicting Risks and Outcomes<br>○ Predict need for procedures or referrals	GPT-4o (OpenAI)	Pediatric cases from unspecified tertiary hospital in Southern Thailand	106 pediatric cases	Traipidok et al,[48] 2025
**Pediatrics Subspecialty**	**Population**	**Pediatrics-Specific Challenge**	**LLM Use Case**	**LLM Use Case Category / Sub-Category / Task***	**LLM Name and Developer**	**Data Source**	**Sample Size**	**Publication**
Developmental-Behavioral Pediatrics	Patients with autism spectrum disorder, developmental language disorder, and global developmental delay	Diagnostic reasoning for complex neurodevelopmental disorders such as autism spectrum disorder remains challenging due to limited tools that can support accurate differential diagnosis from clinical text	Diagnose pediatric developmental conditions	Clinical Decision Support<br>• Diagnostic Decision Support<br>○ Generate differential diagnoses	GPT-4 (OpenAI)	Developmental and behavioral assessment reports, some supplemented with EMR vignettes containing clinical and behavioral details, from Children's Hospital National Clinical Research Center for Child Health and Disorders, Children's Hospital of Chongqing	60 reports	Wei et al,[50] 2023

© 2026 Huang T et al. *JAMA Network Open*.

						Medical University		
**General Pediatrics and Adolescent Medicine**								
General Pediatrics	Random sample from the general pediatric population	Pediatric SDoH elements are primarily documented in unstructured clinical narratives within EHRs	Extract social determinants of health	Clinical Decision Support<br>• Diagnostic Decision Support<br>○ Evaluate social determinants of health	Flan-T5-Large (Google Research), GPT-4 (OpenAI)	Annotated social history sections of clinical notes from University of Washington (UW) hospital system	245 clinical notes[b]	Fu et al,[24] 2024
**Pediatrics Subspecialty**	**Population**	**Pediatrics-Specific Challenge**	**LLM Use Case**	**LLM Use Case Category / Sub-Category / Task***	**LLM Name and Developer**	**Data Source**	**Sample Size**	**Publication**
General Pediatrics	Patients with a documented chief complaint and pediatrician-generated differential diagnosis	Pediatrics has high rates of diagnostic errors and even higher ones in rural settings	Provide pediatric differential diagnoses	Clinical Decision Support<br>• Diagnostic Decision Support<br>○ Generate differential diagnoses	GPT-3 Davinci version (OpenAI)	EHR records, including demographics, symptoms, history, and clinician differential diagnoses from unspecified rural pediatric health care organization in Central Louisiana	150 records[b]	Mansoor et al,[40] 2025
General Pediatrics	Patients and caregivers in our health system	Language barriers in pediatric care can hinder caregiver understanding of discharge instructions, impacting treatment adherence and follow-up[a]	Generate Spanish translations	Patient Communication and Education<br>• Enhancing Accessibility<br>○ Generate visual aids, translate content, make content accessible	GPT-4o (OpenAI)	Source files and previously translated versions of pediatric patient instructions from Boston Children's Hospital	20 patient instruction files	Ray et al,[44] 2025

© 2026 Huang T et al. *JAMA Network Open*.

General Pediatrics (Rare Diseases)	Patients with confirmed diagnoses	Pediatric diagnosis, especially for rare diseases, is difficult and often delayed	Provide differential diagnoses given rare disease clinical cases	Clinical Decision Support<br>• Diagnostic Decision Support<br>○ Generate differential diagnoses	DxGPT (Foundation 29 and Open AI)	Clinical cases, including demographics, clinical presentation, and early diagnostic data from Sant Joan de Déu Barcelona Children's Hospital	70 clinical cases	Alvarez-Estape et al,[17] 2024
**Pediatrics Subspecialty**	**Population**	**Pediatrics-Specific Challenge**	**LLM Use Case**	**LLM Use Case Category / Sub-Category / Task***	**LLM Name and Developer**	**Data Source**	**Sample Size**	**Publication**
General Pediatrics (Rare Diseases)	Patients seen at the Necker Pediatric Hospital	Extracting temporal information from pediatric EHRs (especially for rare diseases) is crucial for diagnosis and treatment planning but challenging due to limited data and variability in language and record formats	Extract temporal entities	Clinical Decision Support<br>• Diagnostic Decision Support<br>○ Recognize disease patterns from symptoms/vitals	Vicuna (Large Model Systems Organization)	EHR record clinical notes describing evolution of rare diseases from Necker Hospital	50 clinical notes	Andrew et al,[18] 2024
Adolescent Medicine	Adolescents who had patient portal accounts at Stanford Children's Health	High rates of guardian access to adolescent patient portals compromise confidentiality and may reduce adolescents' engagement with care, yet current methods to detect such breaches from EHR messages are	Detect guardian authorship and generate responses	Patient Communication and Education<br>• Patient-Provider Messaging<br>○ Share results, draft responses	GPT-4 (OpenAI)	Electronic patient portal messages from Stanford Children's Health	2088 messages	Liang et al,[36] 2024

© 2026 Huang T et al. *JAMA Network Open*.

		limited in accuracy and scalability						
Adolescent Medicine	A random sample of patients from outpatient adolescent encounters	In adolescent care, electronic health records often contain sensitive confidential information in free-text clinical notes that current systems cannot reliably identify or filter, risking unintentional disclosure to guardians and compromising patient confidentiality	Identify confidential content and extract it	Administration and Workflow<br>• Organizing Workflow Processes<br>○ Handle Information requests	GPT-3.5 (OpenAI)	Adolescent progress notes from unspecified outpatient clinic	300 progress notes	Rabbani et al,[43] 2024
**Pediatrics Subspecialty**	**Population**	**Pediatrics-Specific Challenge**	**LLM Use Case**	**LLM Use Case Category / Sub-Category / Task[*]**	**LLM Name and Developer**	**Data Source**	**Sample Size**	**Publication**
Adolescent Medicine	Adolescents seen at Stanford Children's Health	In pediatric care, distinguishing messages sent by adolescent patients versus their parents or guardians is challenging but essential to protect adolescent confidentiality	Identify proxy users, maintain confidentiality in responses, and generate responses	Patient Communication and Education<br>• Patient-Provider Messaging<br>○ Share results, draft responses	GPT-4 (OpenAI)	Adolescent patient portal messages from Stanford Medicine Children's Health	300 messages	Tse et al,[49] 2025

[*]Based on validated LLM clinical use case taxonomy[12]
[a]Challenge inferred by review authors based on study design and context
[b]Model was fine-tuned; however, only the evaluation sample size is reported. Sample sizes used for training and validation during fine-tuning are not included.

© 2026 Huang T et al. *JAMA Network Open*.

© 2026 Huang T et al. *JAMA Network Open*.
