# Supplementary material for: Large Language Models Using Clinical Text in Pediatrics: A Scoping Review
Source: JAMA Netw Open. 2026 Mar 25;9(3):e262443. doi: 10.1001/jamanetworkopen.2026.2443 (PMC13019234; doi:10.1001/jamanetworkopen.2026.2443)
Supplement: Supplement 2. — Data Sharing Statement [file jamanetwopen-e262443-s002.pdf]

Data Sharing Statement

Huang. Large Language Models Using Clinical Text in Pediatrics. *JAMA Netw Open*. Published March 25, 2026. doi:10.1001/jamanetworkopen.2026.2443

Data
Data available: No
